# *Drosophila centrocortin* is dispensable for centriole duplication but contributes to centrosome separation

**DOI:** 10.1093/g3journal/jkab434

**Published:** 2021-12-23

**Authors:** Dipen S Mehta, Hala Zein-Sabatto, Pearl V Ryder, Jina Lee, Dorothy A Lerit

**Affiliations:** 1 College of Science and Mathematics, Augusta University, Augusta, GA 30912, USA; 2 Department of Cell Biology, Emory University School of Medicine, Atlanta, GA 30322, USA; 3 Wandrer, Atlanta, GA 30340, USA; 4 Emory College of Arts and Sciences, Emory University, Atlanta, GA 30322, USA

**Keywords:** centrosome, mitosis, RNA localization, centrocortin

## Abstract

Centrosomes are microtubule-organizing centers that duplicate exactly once to organize the bipolar mitotic spindle required for error-free mitosis. Prior work indicated that *Drosophila centrocortin* (*cen*) is required for normal centrosome separation, although a role in centriole duplication was not closely examined. Through time-lapse recordings of rapid syncytial divisions, we monitored centriole duplication and the kinetics of centrosome separation in control vs *cen* null embryos. Our data suggest that although *cen* is dispensable for centriole duplication, it contributes to centrosome separation.

## Introduction

Centrosomes are the microtubule-organizing centers (MTOCs) of most eukaryotic cells and promote error-free mitosis through organization of the bipolar mitotic spindle. Centrosome function as a MTOC is instructed by the pericentriolar material (PCM), a matrix of proteins that encircles the central pair of centrioles (Nigg and Raff 2009).

Centrosomes are essential for early *Drosophila* embryogenesis, where they coordinate rapid S-M abridged nuclear division cycles and cortical migration of the nuclei to give rise to the syncytial blastoderm embryo (Rothwell and Sullivan 2000; Stevens et al. 2007; Gonzalez 2008). As conserved in many organisms, *Drosophila* embryogenesis requires a maternal supply of mRNAs and proteins to support early development until zygotic genome activation; thus, syncytial-stage embryos are largely transcriptionally quiescent (Tadros and Lipshitz 2009). The early embryonic mitotic divisions, therefore, are predominantly regulated by maternally endowed supplies.

Among the resident components of centrosomes are mRNAs (Marshall and Rosenbaum 2000; Alliegro 2011). A genome-wide screen within early *Drosophila* embryos identified a subset of transcripts enriched near spindle poles and encoding proteins key to centrosome function and mitotic progression (Lécuyer et al. 2007). The localization of mRNAs to centrosomes is evolutionarily conserved and observed across taxa (Groisman et al. 2000; Lambert and Nagy 2002; Alliegro et al. 2006; Blower et al. 2007; Lécuyer et al. 2007; Sepulveda et al. 2018; Zein-Sabatto and Lerit 2021). Despite this high level of conservation, functions of centrosome-localized mRNAs remain underexplored.

Systematic functional analysis of a few individual mRNAs suggests local RNAs support spindle morphogenesis and mitotic progression, as demonstrated for localized *cyclin B* mRNA in *Xenopus* oocytes (Groisman et al. 2000). In *Drosophila*, recent work indicates *centrocortin* (*cen*) mRNA localized to centrosomes is similarly required for error-free mitosis. Here, the *cen* coding sequence is necessary and sufficient for *cen* mRNA localization to pericentrosomal RNA granules, which likely function as sites of translational regulation (Bergalet et al. 2020; Ryder et al. 2020). Indeed, high-throughput analyses support an emerging view that co-translational transport supports the localization of several conserved centrosome mRNAs (Sepulveda et al. 2018; Chouaib et al. 2020; Kwon et al. 2021; Safieddine et al. 2021).

Historically, several hypotheses were suggested to account for why mRNAs reside at centrosomes, including the postulation that mRNA may instruct centrosome duplication (Alliegro et al. 2006). In this view, mRNA may serve as the genetic material required to support the semi-conservative replication of centrioles, where an older “mother” centriole serves as the template for the younger “daughter” centriole (Pelletier and Yamashita 2012). Alternatively, mRNA may support the structural integrity of the centrosome (Woodruff et al. 2018). Other models of centrosomal mRNA functions are also possible (Ryder and Lerit 2018). Importantly, these models remain to be tested.

In this study, we investigated whether *cen* contributes to centriole duplication. Normally, centrioles duplicate only once per cell cycle, and this regulation is key to prevent multipolar spindles and chromosomal instability (Wong and Stearns 2003; Tsou and Stearns 2006). In *Drosophila*, *cen* mutant embryos show mitotic defects, including errant centrosome separation and multipolar spindles, despite normal microtubule assembly (Kao and Megraw 2009). However, centriole duplication was not previously examined in *cen* mutants. Given the requirement for local *cen* mRNA for the integrity of centrosome functions, we assessed the requirement of *cen* in centriole duplication and centrosome separation in live embryos.

## Materials and methods

### Fly stocks

*Drosophila* strains used in this study are summarized in Table  1.

**Table 1:** *Drosophila* strains used.

*Drosophila* strain	Source	Description
*PBAC-GFP-Cnn*	Lerit et al. (2015)	Cnn tagged at the N-terminus with EGFP under endogenous regulatory elements
*P[Ubi-RFP-PACT]*	This paper	N-terminal fusion of RFP to a C-terminal fragment of PLP containing the PACT domain
*cen* ^f04787^	Kao and Megraw (2009)	Protein and RNA null allele; Bloomington stock #18805

Flies were raised on Bloomington formula “Fly Food B” (LabExpress; Ann Arbor, MI, USA), and crosses were maintained at 25°C in a light and temperature-controlled incubator chamber. To examine maternal effects, *cen* mutant embryos are progeny derived from homozygous *cen^f04787^* mutant mothers.

### Generation of RFP-PACT

*P[Ubi-RFP-PACT]* was a gift from Nasser Rusan (NIH) and was generated by introducing amino acids 2,479–3,555 from PLP-PF (Fragment 5), into the vector *pURW* (plasmid 1282, *Drosophila Genomics Resource Center*) to generate an RFP fusion, as described for the GFP fusions in Galletta et al. (2014). Transgenic animals were generated by BestGene, Inc. (Chino Hills, CA, USA).

### Microscopy

For live imaging, dechorionated 1–2 h embryos were adhered to a 22 × 30 mm #1.5 coverslip using glue extracted from double-sided tape (3M), covered with Halocarbon oil 700 (H8898; Millipore-Sigma), and inverted onto a 50-mm gas permeable dish (Sarstedt) with broken #1 coverslips used as spacers. Images were captured on a Nikon Ti-E inverted microscope fitted with Yokagawa CSU-X1 spinning disk head using a motorized stage, Nikon LU-N4 solid-state laser launch (15 mW 405, 488, 561, and 647 nm), Hamamatsu Orca Flash 4.0 v2 digital CMOS camera, and a Nikon 100x, 1.49 NA Apo TIRF oil immersion objective. Images were acquired at 300 ms exposure times over 0.5 μm Z-intervals through 3.5 μm of tissue at 20 s time intervals for one or more complete embryonic nuclear cycles (NCs).

### Image analysis

Images were randomized, and the experimenter was blinded to genotype during analysis. We measured the time elapsed from when centrioles were duplicated, visible as 2 proximal RFP-PACT signals, until they were fully separated. We define full separation as the moment when centrioles remain at distal sides of the nucleus, approximately late prophase. Time-lapse recordings from age-matched nuclear division cycle 13 samples were used. Once centrosomes were fully separated, the distance between centrosomes was measured using the line tool. The time between successive centriole duplication events was monitored between NCs 12 and 13. Image analysis was completed in FIJI (NIH; Schindelin et al. 2012). Images were assembled using Adobe Photoshop and Illustrator software.

### Statistical analysis

Data were unblinded, plotted, and statistical analysis was performed using Microsoft Excel and GraphPad Prism software. Normal data distribution was tested using a D’Agostino and Pearson normality test, followed by a 2-tailed nonparametric Mann–Whitney test, 2-tailed *t*-test with Welch’s correction, or a chi-square test, as indicated in the figure legend. Data shown are representative results from at least 2 independent experiments.

## Results and discussion

To address whether *cen* contributes to centriole duplication, we examined the kinetics of centrosome duplication and separation in live control *vs* *cen* mutant *Drosophila* embryos. For these studies, we used embryos collected from homozygous *cen*^f04787^ mothers. The *cen*^f04787^ allele is a *PBac* transposon insertion disrupting the *cen* locus (Bellen et al. 2011). Prior work confirms *cen*^f04787^ animals are null for Cen protein (Kao and Megraw 2009) and mRNA (Bergalet et al. 2020).

**Figure 1:**
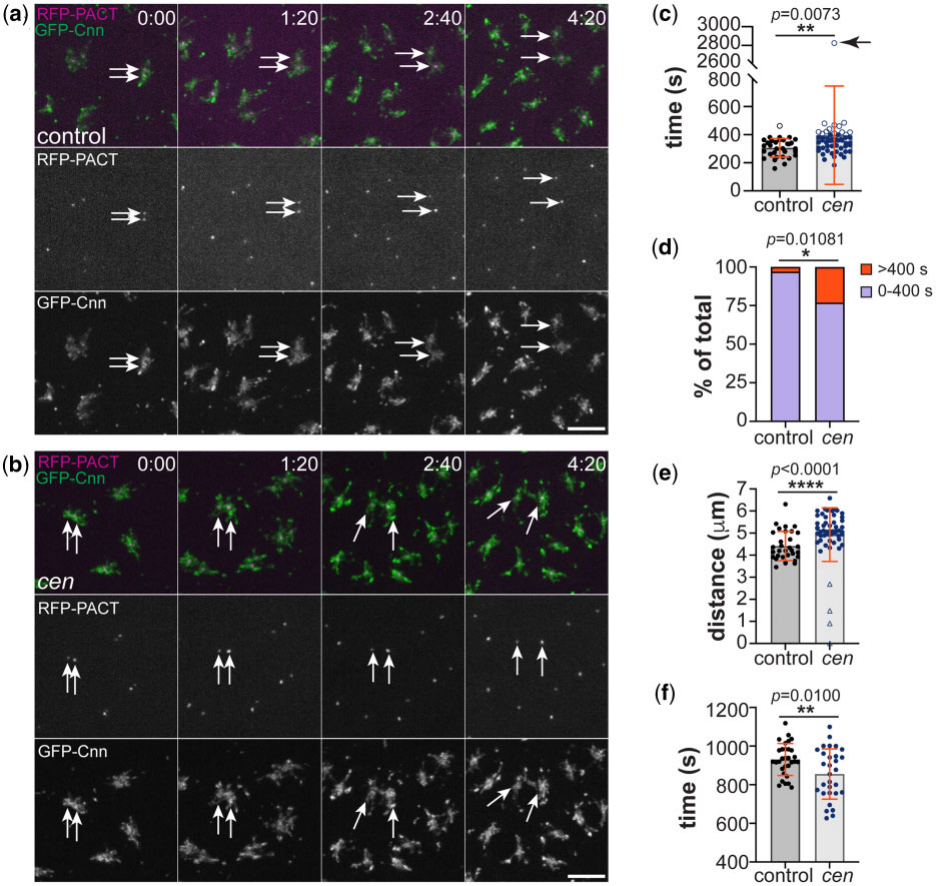
*cen* promotes centrosome separation. Individual timepoints from time-lapse recordings of NC 13 a) control and b) *cen* mutant embryos expressing *RFP-PACT* (magenta) and *GFP-Cnn* (green). Time is shown in minutes: seconds. Stills are from Supplementary Videos 1 and 2. Arrows mark pairs of separating centrosomes. c) Quantification shows the time (s) required to fully separate the duplicated centrosomes from control vs *cen* mutant NC 13 embryos. Each dot represents a single measurement from WT: *N* = 32 centrosomes from 2 embryos and *cen*: *N* = 52 centrosomes from 3 embryos. Open dots represent centrosomes that required >400 s to separate; the arrow marks a centrosome pair that failed to separate prior to the end of the recording. **P = 0.0073 by a 2-tailed Mann–Whitney test. d) Distribution analysis of the time to complete centrosome separation, as detailed in (c). A chi-square test indicates that centrosomes from *cen* mutant embryos take significantly longer time (>400 s) to separate relative to controls; *X*^2^ (1, *N* = 86) = 6.4961, *P = 0.0108. e) Distance (µm) measured between NC 13 centrosomes at the end point of separation, as in (c); ****P < 0.0073 by a 2-tailed Mann–Whitney test. f) Quantification of the time (s) elapsed between centriole duplication at NC 12 and NC 13 from WT: *N* = 31 centrioles from 2 embryos and *cen*: *N* = 30 centrioles from 2 embryos. **P = 0.0100 by *t*-test with Welch’s correction. Bars, 5 μm.

NC 13 control and *cen* mutant embryos co-expressing *GFP-Cnn* to label the PCM and *RFP-PACT* to mark the centrioles were monitored by video microscopy (Fig. 1, a and b, Supplementary Videos 1 and 2). Through blinded image analysis, we calculated the time elapsed from when centrosomes are duplicated, visible as 2 proximal RFP-PACT signals, until they were fully separated (Fig. 1c). Similar to controls, centrosomes from *cen* null embryos duplicated and initiated centriole separation. However, about 20% of *cen* centrosomes required more time to fully separate (12/52 centrosome pairs; open dots, Fig. 1c) or failed to separate (arrow), consistent with prior work (Kao and Megraw 2009). On average, centrosomes from NC 13 *cen* mutant embryos required about 30% more time than controls to separate (time to separate, expressed as mean ± SD, was 396.1 ± 350.1 s for *cen* mutants *vs* 306.3 ± 65.8 s for controls; *P = *0.0073 by Mann–Whitney test). Examination of the frequency distribution of centrosome separation velocities confirmed that the centrosomes in *cen* mutant embryos require more time than controls (>400 s) to separate (Fig. 1d; *P = 0.01081 by chi-square test). These data indicate *cen* is required to complete efficient centrosome separation.

Our data show Cen supports the kinetics of centrosome separation. We next measured the distance between centrosomes once fully separated (Fig. 1, a and b; 4:20) to further investigate the extent of centrosome separation in *cen* mutant embryos. On average, control centrosomes were separated 4.4 ± 0.7 µm in NC 13 late prophase embryos (Fig. 1e). In contrast, a subset of *cen* mutant centrosomes failed to separate or separated less than 3 µm (7.5%, *N* = 4/53; Fig. 1e, open symbols). Most *cen* mutant centrosomes, however, did successfully separate. Unexpectedly, many *cen* mutant centrosomes separated greater distances than controls, perhaps due to centrosome detachment errors.

To determine if *cen* is required for centriole duplication, we quantified the time between successive centriole duplication events, spanning NC 12 to 13. All examined centrioles completed duplication, indicating that *cen* is dispensable for centriole duplication (Fig. 1f). A small proportion of *cen* mutant centrioles duplicated at faster rates than controls (Fig. 1f). While the underlying mechanism of this defect remains unknown, these more rapidly dividing centrioles likely correspond to anucleated centrosomes following the ejection of damaged nuclei from the syncytial blastoderm cortex. Taken together, our analysis indicates *cen* contributes to normal centrosome separation kinetics but is not required for centriole duplication. Further work is required to unearth precisely how *cen* promotes centrosome separation.

Although several mRNAs localize to centrosomes, the functions of most centrosome proximal mRNAs remain unstudied (Zein-Sabatto and Lerit 2021). *Drosophila cen* mRNA is a notable exception, as mislocalization of *cen* mRNA to the anterior cortex via the *bicoid* (*bcd*) 3’-UTR demonstrated that local *cen* mRNA is required to recruit Cen protein to distal centrosomes and support spindle morphogenesis, genome stability, and embryonic viability (Ryder et al. 2020). Among the phenotypes noted in that study were mispositioned centrosomes, which could be an indirect effect of the large-scale nuclear fallout DNA damage response observed in *cen-bcd-3’UTR* embryos. To circumvent this technical challenge, we examined centrosome separation in *cen* null embryos, which lack mRNA and protein. From this study, we conclude that local *cen* activity is dispensable for normal centriole duplication but supports centrosome separation. Whether local *cen* mRNA or protein is sufficient to support centrosome separation remains to be determined.

## Data availability

Strains are available upon request. The authors affirm that all data necessary for confirming the conclusions of the article are present within the article, figures, and tables. Supplemental data are available on Figshare: time-lapse recordings Supplementary Video 1 (control) and Supplementary Video 2 (*cen* mutant) show embryos expressing *RFP-PACT* and *GFP-Cnn*. Supplemental Material included at figshare: https://doi.org/10.25387/g3.17096684.

## References

[jkab434-B1] Alliegro MC. The centrosome and spindle as a ribonucleoprotein complex. Chromosome Res. 2011;19(3):367–376. 21287260 10.1007/s10577-011-9186-7

[jkab434-B2] Alliegro MC , Alliegro MA, Palazzo RE. Centrosome-associated RNA in surf clam oocytes. Proc Natl Acad Sci USA. 2006;103(24):9034–9038. 16754862 10.1073/pnas.0602859103 PMC1482561

[jkab434-B3] Bellen HJ , Levis RW, He Y, Carlson JW, Evans-Holm M, Bae E, Kim J, Metaxakis A, Savakis C, Schulze KL, et al The *Drosophila* gene disruption project: progress using transposons with distinctive site specificities. Genetics. 2011;188(3):731–743. 21515576 10.1534/genetics.111.126995 PMC3176542

[jkab434-B4] Bergalet J , Patel D, Legendre F, Lapointe C, Benoit Bouvrette LP, Chin A, Blanchette M, Kwon E, Lécuyer E. Inter-dependent centrosomal co-localization of the *cen* and *ik2* cis-natural antisense mRNAs in *Drosophila*. Cell Rep. 2020;30(10):3339–3352.e3336. 32160541 10.1016/j.celrep.2020.02.047

[jkab434-B5] Blower MD , Feric E, Weis K, Heald R. Genome-wide analysis demonstrates conserved localization of messenger RNAs to mitotic microtubules. J Cell Biol. 2007;179(7):1365–1373. 18166649 10.1083/jcb.200705163 PMC2373496

[jkab434-B6] Chouaib R , Safieddine A, Pichon X, Imbert A, Kwon OS, Samacoits A, Traboulsi A-M, Robert M-C, Tsanov N, Coleno E, et al A dual protein-mRNA localization screen reveals compartmentalized translation and widespread co-translational RNA targeting. Dev Cell. 2020;54(6):773–791.e775. 32783880 10.1016/j.devcel.2020.07.010

[jkab434-B7] Galletta BJ , Guillen RX, Fagerstrom CJ, Brownlee CW, Lerit DA, Megraw TL, Rogers GC, Rusan NM. *Drosophila* pericentrin requires interaction with calmodulin for its function at centrosomes and neuronal basal bodies but not at sperm basal bodies. Mol Biol Cell. 2014;25(18):2682–2694. 25031429 10.1091/mbc.E13-10-0617 PMC4161505

[jkab434-B8] Gonzalez C. Centrosome function during stem cell division: the devil is in the details. Curr Opin Cell Biol. 2008;20(6):694–698. 18996192 10.1016/j.ceb.2008.10.003

[jkab434-B9] Groisman I , Huang Y-S, Mendez R, Cao Q, Theurkauf W, Richter JD. CPEB, maskin, and *cyclin B1* mRNA at the mitotic apparatus: implications for local translational control of cell division. Cell. 2000;103(3):435–447. 11081630 10.1016/s0092-8674(00)00135-5

[jkab434-B10] Kao LR , Megraw TL. Centrocortin cooperates with centrosomin to organize *Drosophila* embryonic cleavage furrows. Curr Biol. 2009;19(11):937–942. 19427213 10.1016/j.cub.2009.04.037 PMC2714769

[jkab434-B11] Kwon OS , Mishra R, Safieddine A, Coleno E, Alasseur Q, Faucourt M, Barbosa I, Bertrand E, Spassky N, Le Hir H, et al Exon junction complex dependent mRNA localization is linked to centrosome organization during ciliogenesis. Nat Commun. 2021;12(1):1351. 33649372 10.1038/s41467-021-21590-w PMC7921557

[jkab434-B12] Lambert JD , Nagy LM. Asymmetric inheritance of centrosomally localized mRNAs during embryonic cleavages. Nature. 2002;420(6916):682–686. 12478296 10.1038/nature01241

[jkab434-B13] Lécuyer E , Yoshida H, Parthasarathy N, Alm C, Babak T, Cerovina T, Hughes TR, Tomancak P, Krause HM. Global analysis of mRNA localization reveals a prominent role in organizing cellular architecture and function. Cell. 2007;131(1):174–187. 17923096 10.1016/j.cell.2007.08.003

[jkab434-B14] Lerit DA , Jordan HA, Poulton JS, Fagerstrom CJ, Galletta BJ, Peifer M, Rusan NM. Interphase centrosome organization by the PLP-Cnn scaffold is required for centrosome function. J Cell Biol. 2015;210(1):79–97. 26150390 10.1083/jcb.201503117 PMC4494003

[jkab434-B15] Marshall WF , Rosenbaum JL. Are there nucleic acids in the centrosome? Curr Top Dev Biol. 2000;49:187–205. 11005019 10.1016/s0070-2153(99)49009-x

[jkab434-B16] Nigg EA , Raff JW. Centrioles, centrosomes, and cilia in health and disease. Cell. 2009;139(4):663–678. 19914163 10.1016/j.cell.2009.10.036

[jkab434-B17] Pelletier L , Yamashita YM. Centrosome asymmetry and inheritance during animal development. Curr Opin Cell Biol. 2012;24(4):541–546. 22683192 10.1016/j.ceb.2012.05.005 PMC3425708

[jkab434-B18] Rothwell WF , Sullivan W. The centrosome in early *Drosophila* embryogenesis. Curr Top Dev Biol. 2000;49:409–447. 11005030 10.1016/s0070-2153(99)49020-9

[jkab434-B19] Ryder PV , Fang J, Lerit DA. *centrocortin* RNA localization to centrosomes is regulated by FMRP and facilitates error-free mitosis. J Cell Biol. 2020;219:e202004101. 33196763 10.1083/jcb.202004101 PMC7716377

[jkab434-B20] Ryder PV , Lerit DA. RNA localization regulates diverse and dynamic cellular processes. Traffic. 2018;19(7):496–502. 29653028 10.1111/tra.12571 PMC6003861

[jkab434-B21] Safieddine A , Coleno E, Salloum S, Imbert A, Traboulsi A-M, Kwon OS, Lionneton F, Georget V, Robert M-C, Gostan T, et al A choreography of centrosomal mRNAs reveals a conserved localization mechanism involving active polysome transport. Nat Commun. 2021;12(1):1352. 33649340 10.1038/s41467-021-21585-7 PMC7921559

[jkab434-B22] Schindelin J , Arganda-Carreras I, Frise E, Kaynig V, Longair M, Pietzsch T, Preibisch S, Rueden C, Saalfeld S, Schmid B, et al Fiji: an open-source platform for biological-image analysis. Nat Methods. 2012;9(7):676–682. 22743772 10.1038/nmeth.2019 PMC3855844

[jkab434-B23] Sepulveda G , Antkowiak M, Brust-Mascher I, Mahe K, Ou T, Castro NM, Christensen LN, Cheung L, Jiang X, Yoon D, et al Co-translational protein targeting facilitates centrosomal recruitment of PCNT during centrosome maturation in vertebrates. eLife. 2018;7:e34959. 29708497 10.7554/eLife.34959 PMC5976437

[jkab434-B24] Stevens NR , Raposo AA, Basto R, St Johnston D, Raff JW. From stem cell to embryo without centrioles. Curr Biol. 2007;17(17):1498–1503. 17716897 10.1016/j.cub.2007.07.060 PMC1971134

[jkab434-B25] Tadros W , Lipshitz HD. The maternal-to-zygotic transition: a play in two acts. Development. 2009;136(18):3033–3042. 19700615 10.1242/dev.033183

[jkab434-B26] Tsou MF , Stearns T. Mechanism limiting centrosome duplication to once per cell cycle. Nature. 2006;442(7105):947–951. 16862117 10.1038/nature04985

[jkab434-B27] Wong C , Stearns T. Centrosome number is controlled by a centrosome-intrinsic block to reduplication. Nat Cell Biol. 2003;5(6):539–544. 12766773 10.1038/ncb993

[jkab434-B28] Woodruff JB , Hyman AA, Boke E. Organization and function of non-dynamic biomolecular condensates. Trends Biochem Sci. 2018;43(2):81–94. 29258725 10.1016/j.tibs.2017.11.005

[jkab434-B29] Zein-Sabatto H , Lerit DA. The identification and functional analysis of mRNA localizing to centrosomes. Front Cell Dev Biol. 2021;9:782802. 34805187 10.3389/fcell.2021.782802 PMC8595238

